# Supplementary material for: Mechanisms governing the pioneering and redistribution capabilities of the non-classical pioneer PU.1
Source: Nat Commun. 2020 Jan 21;11:402. doi: 10.1038/s41467-019-13960-2 (PMC6972792; doi:10.1038/s41467-019-13960-2)
Supplement: Supplementary file 7 — Source data [file 41467_2019_13960_MOESM7_ESM.zip › Source_Data/Figure6/Figure6B_MotifScanOutput/ETS1_mutPU.1/homerResults/motif9.similar.html]

motif9

## Information for motif9

C
T
A
G
G
C
A
T
A
G
C
T
A
T
G
C
G
A
C
T
G
C
A
T
C
G
T
A
C
G
A
T
A
G
T
C
C
G
A
T
A
T
C
G
  
Reverse Opposite:  

T
A
G
C
C
G
T
A
A
C
T
G
C
G
T
A
A
C
G
T
C
G
T
A
C
T
G
A
T
A
C
G
T
C
G
A
C
G
T
A
G
A
T
C
  

|  |  |
| --- | --- |
| p-value: | 1e-187 |
| log p-value: | -4.328e+02 |
| Information Content per bp: | 1.583 |
| Number of Target Sequences with motif | 2050.0 |
| Percentage of Target Sequences with motif | 15.20% |
| Number of Background Sequences with motif | 2238.5 |
| Percentage of Background Sequences with motif | 6.38% |
| Average Position of motif in Targets | 98.7 +/- 52.4bp |
| Average Position of motif in Background | 101.0 +/- 62.2bp |
| Strand Bias (log2 ratio + to - strand density) | 0.1 |
| Multiplicity (# of sites on avg that occur together) | 1.10 |
| Motif File: | file (matrix) reverse opposite |

### Similar de novo motifs found

|  |  |  |  |  |  |  |  |
| --- | --- | --- | --- | --- | --- | --- | --- |
| Rank | Match Score | Redundant Motif | P-value | log P-value | % of Targets | % of Background | Motif file |
| 1 | 0.967 | A C G T A T G C G C A T C G A T C T G A G C A T A G T C G C A T T A C G | 1e-184 | -424.807704 | 15.79% | 6.85% | motif file (matrix) |
| 2 | 0.955 | A C G T A G T C A C G T A C G T C G T A A C G T A G T C C G A T A T C G G A C T | 1e-178 | -412.010241 | 14.27% | 5.92% | motif file (matrix) |
| 3 | 0.936 | C A G T G T A C G C A T A C T G A G C T A C G T C G A T C G T A A C G T A G T C G C A T C A G T A C G T | 1e-173 | -400.062761 | 17.75% | 8.45% | motif file (matrix) |
| 4 | 0.935 | G C T A A C T G C G T A A C G T C G T A C G T A T C A G | 1e-166 | -384.316653 | 19.01% | 9.53% | motif file (matrix) |
| 5 | 0.955 | A C T G G C A T A G C T A G T C A C G T A C G T C G T A A C G T A G T C C G A T A T C G G A C T | 1e-166 | -383.920259 | 13.13% | 5.39% | motif file (matrix) |
| 6 | 0.927 | C T G A T A G C C G T A A C T G C G T A A C G T C G T A C G T A | 1e-152 | -350.674857 | 17.12% | 8.48% | motif file (matrix) |
| 7 | 0.767 | G T A C C G A T T A C G G T C A A C G T A T C G G A C T G A C T C G T A A C G T A G T C C G A T T A C G G C T A | 1e-37 | -87.306429 | 1.55% | 0.38% | motif file (matrix) |
